# Assessment of Oral Vancomycin-Induced Alterations in Gut Bacterial Microbiota and Metabolome of Healthy Men

**DOI:** 10.3389/fcimb.2021.629438

**Published:** 2021-05-27

**Authors:** Andrew HyoungJin Kim, Yujin Lee, Eunwoo Kim, Sang Chun Ji, Jae-Yong Chung, Joo-Youn Cho

**Affiliations:** ^1^ Department of Clinical Pharmacology and Therapeutics, Seoul National University College of Medicine and Hospital, Seoul, South Korea; ^2^ Kidney Research Institute, Seoul National University Medical Research Center, Seoul, South Korea; ^3^ Department of Clinical Pharmacology and Therapeutics, Seoul National University College of Medicine and Bundang Hospital, Seongnam, South Korea; ^4^ Department of Biomedical Sciences, Seoul National University College of Medicine, Seoul, South Korea

**Keywords:** vancomycin, gut bacterial microbiota, metabolome, metabolomics, dysbiosis-related disease, drug metabolism

## Abstract

Several classes of antibiotics have reduced the mortality caused by infectious diseases; however, orally administered antibiotics alter the composition of gut microbiota, leading to dysbiosis-related disease. Therefore, in this study, we used 16S rRNA gene sequencing- and metabolomics-based approaches to investigate the effects of oral vancomycin on gut bacterial microbiota and the metabolome in biospecimens collected from healthy men. Samples collected from 11 healthy men were analyzed using 16S rRNA gene sequencing and metabolomics. 16S rRNA gene sequencing was performed to analyze the gut bacterial microbiota, and GC-TOFMS-based untargeted metabolomics was performed to analyze fecal, urine, and plasma metabolomics. Spearman’s rank correlation was utilized to explore the associations between gut bacterial microbiota and metabolome. Fecal 16S rRNA gene sequencing analysis showed decreased relative abundance of genera belonging to the phyla Bacteroidetes and Firmicutes, and increased relative abundance of genera of the phyla Proteobacteria and Fusobacteria. Fecal metabolomics analysis showed that levels of uracil, L-aspartic acid, lithocholic acid, and deoxycholic acid were significantly higher at baseline, whereas that of dihydrouracil was significantly higher after vancomycin administration. No significant metabolic markers were selected from urine and plasma metabolomics analysis. This study demonstrates that oral vancomycin administration induces alterations in gut bacterial microbiota and metabolome. Correlation analysis between our two datasets shows that alteration of the gut bacterial microbiota, induced by oral vancomycin, potentially affected the systemic activity of dihydropyrimidine dehydrogenase. This correlation should be further examined in future studies to define the effects of gut bacterial microbiota on drug-metabolizing enzymes, thereby contributing to the development of personalized therapy.

## Introduction

The discovery of the first true antibiotic, penicillin, in 1928 has greatly contributed to the decline of mortality caused by infectious diseases (Fleming, 1980; Armstrong et al., 1999). Since then, various classes of antibiotics, such as tetracyclines, β-lactams, and glycopeptide antibiotics have been developed and used for the treatment of different infectious diseases (Macias, 1973; Bradford and Jones, 2012; Butler et al., 2014). Among the glycopeptide class of antibiotics, vancomycin is extensively used for the treatment of infections caused by gram-positive pathogens such as methicillin-resistant *Staphylococcus aureus* (MRSA) and the gut anaerobe, *Clostridioides difficile* (Gordon and Lowy, 2008; Leffler and Lamont, 2015). Although vancomycin can exert several beneficial effects against specific infections, oral vancomycin can also cause negative effects such as gut dysbiosis resulting in significant alteration of gut bacterial composition along with important endogenous metabolites such as bile acids (Vrieze et al., 2014; Isaac et al., 2017).

Gut microbiota is a term used to describe microbial communities within the gastrointestinal tract of an organism (Bäckhed et al., 2005; Neish, 2009), and plays important roles in human health by protecting the body against pathogens and regulating the gut immune system. Therefore, the maintenance of healthy gut microbiota is considered essential in the treatment of a wide range of diseases including cancer, metabolic disorders, and inflammatory diseases (Turnbaugh et al., 2006; Stecher and Hardt, 2008; Wang et al., 2012; Zhu et al., 2013; Min and Rhee, 2015; Lloyd-Price et al., 2016; Duvallet et al., 2017). Gut microbiota also exert various direct and indirect effects on the pharmacokinetics (PK) of certain drugs such as lovastatin, digoxin, and acetaminophen potentially resulting in variation in their therapeutic efficacy (Clayton et al., 2009; Jourova et al., 2016; Wilson and Nicholson, 2017). Accordingly, gut microbiota can directly contribute to the biotransformation of prodrugs, or indirectly contribute to increase or decrease the hepatic metabolism by interacting with several xenobiotics metabolism pathways such as the pregnane X receptor (PXR) mediated xenobiotics sensing pathway. Previous *in vitro* studies have shown the regulating effect of secondary bile acids (mainly lithocholic acid) on the major drug-metabolizing enzyme, cytochrome P450 3A subfamily (CYP3A), through PXR activation (De Wildt et al., 1999; Ridlon et al., 2006; Kakiyama et al., 2013; Sayin et al., 2013; Zanger and Schwab, 2013). Moreover, secondary bile acids are mainly biotransformed by gut bacterial species and previous studies have shown that oral administration of vancomycin alters fecal bile acids concentration. Therefore, we hypothesized that oral vancomycin-induced dysbiosis of the gut microbiota could cause the disruption of CYP3A activity and that this could be evaluated through the analysis of CYP3A activity and expression using well-known and validated metabolic markers described in previous studies (Lee et al., 2017; Kim et al., 2018b; Kim et al., 2018a; Lee et al., 2021). Since antibiotics are extensively used in various disease, screening of the gut bacteria indirectly responsible for affecting CYP3A activity will provide valuable insight for the development of personalized medicine in the future (Behrouzi et al., 2019).

Metabolomics is a comprehensive analysis of small molecules (metabolites) in a biological specimen (Nicholson and Lindon, 2008). Because metabolites are the end products and intermediates of cellular regulatory processes, they reflect the phenotypical variations taking place in live organisms (Fiehn, 2002), and, thus, has been used in various fields including agriculture, the study of human disease, and medicine (Dixon et al., 2006; Lewis et al., 2008; Trock, 2011; Wishart, 2016). Unsurprisingly, metabolomics has also been used to define the role of the gut microbiota in human health (Nicholson et al., 2012; Marcobal et al., 2013; Vernocchi et al., 2016; Luan et al., 2019). Gut microbiota plays critical roles in human health by producing metabolites such as bile acids, choline, and short-chain fatty acids, which further contribute to the regulation of gut immunity (Kim, 2018). Therefore, a combined analysis using 16S rRNA gene sequencing and metabolomics could allow us to determine the effects of gut microbiota on human health, and the mechanisms of action involved in this activity. In this study, we investigated the effects of oral vancomycin on gut bacterial microbiota and the metabolome through the analysis of biospecimens collected from healthy men using 16S rRNA gene sequencing and metabolomics approaches. Further, we assessed the correlation between the potential effect of vancomycin-induced gut microbiota-dysbiosis and drug.

## Materials and Methods

### Study Design

**Figure 1:**
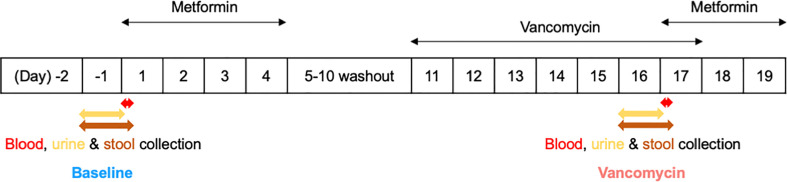
Simplified clinical trial schema.

This study was part of a phase I clinical trial approved by the Institutional Review Board of Seoul National University Bundang Hospital (IRB No. B-1809-492-003). The overall trial was conducted with an open-label, single-arm design to investigate the effects of the gut microbiome on the pharmacokinetics, pharmacodynamics, and safety of metformin in healthy men. Written informed consent was obtained from all participants prior to participation in the study. A total of 15 healthy males aged 19–45 years, weighing 50–100 kg and with a BMI of 18.0–28.0 kg/m^2^, were enrolled in this clinical trial. Subjects with clinically significant diseases or history of liver and kidney disease, as well as, immune, digestive, endocrine, cardiovascular diseases, among others, were excluded from the study. In addition, subjects who have taken prescription drugs, including antibiotics, within 2 weeks before the first dose, or who have taken over-the-counter drugs within 10 days before the first dose, were also excluded from this clinical trial. During the hospitalization period of the clinical trial, the subjects received standardized meals that did not contain lactic acid bacteria. The subjects were asked to consume the entire amount of meals, and meals other than those provided were prohibited. This study, which is a part of the mentioned clinical trial, was conducted to analyze the effects of oral vancomycin on the composition of the gut bacterial microbiota of 11 healthy men (**Figure 1**). Subjects enrolled in this study received 1000 mg of metformin HCl (oral metformin) twice daily from day 1 to day 4, where the first dose was adjusted to 500 mg of oral metformin for the safety of subjects. After a 5-day washout period from days 5–10, the subjects received 500 mg of vancomycin HCl (oral vancomycin) twice daily from days 11–17, where the first dose was adjusted to 250 mg of oral vancomycin for safety of the subjects.

### Sample Collection

For CYP3A4 mRNA analysis, 5 mL of blood was collected in a PAXgene blood RNA tube from PreAnalytiX (Hombrechtikon, Switzerland). To obtain plasma, 6 mL of blood was collected in heparin tubes and centrifuged at 4°C, 2000 x*g* for 10 mins. In both cases, blood was collected 30 minutes after blood collection for plasma metabolomic analysis, on days 1 and 17 before the administration of oral metformin.

Urine was collected on days -1 and 16 before oral administration of metformin in a 12-hour interval for metabolomic analysis. Stool samples from 24 h were collected in sterile plastic bags and homogenized using EZLAB WH4000-2751-9 Bag Mixer (Yongin, Korea) from days -1 to 1 and 16 to 17 before metformin administration. Samples collected between days -1 and 1 were designated as Baseline, and those collected from days 16 to 17 as Vancomycin. These samples were collected from all participants and stored frozen at below -70°C until analysis.

### Chemicals

The fatty acid methyl ester mixture (FAME) used as an internal standard, and the reference standards used for identification of the selected markers were obtained from Sigma-Aldrich (St. Louis, MO). HPLC grade isopropanol, acetonitrile, and water used for sample extraction were purchased from J.T. Baker Chemical Co (Phillipsburg, NJ). Pyridine, methoxamine hydrochloride (MeOX), and *N*-methyl-*N*-(trimethylsilyl)trifluoroacetamide (MSTFA) used for derivatization were purchased from Sigma-Aldrich (St. Louis, MO).

### Fecal 16S rRNA Gene Sequencing Analysis

DNA was extracted using the PowerSoil^®^ DNA Isolation Kit (MO BIO, Carlsbad, CA) according to the manufacturer’s protocol. DNA quantification was performed with PicoGreen (Invitrogen, Carlsbad, CA) and DNA quality was evaluated using a Nanodrop spectrophotometer. Each sequenced sample was prepared according to the Illumina 16S Metagenomic Sequencing Library protocols. The 16S *rRNA* genes were amplified using the 16S V3–V4 primers: 16S Amplicon PCR Forward Primer 341F (5’ TCGTCGGCAGCGTCAGATGTGTATAAGAGACAGCCTACGGGNGGCWGCAG) and 16S Amplicon PCR Reverse Primer 805R (5’ GTCTCGTGGGCTCGGAGATGTGTATAAGAGACAGGACTACHVGGGTATCTAATCC) (Herlemann et al., 2011).

gDNA (2 ng) was amplified using PCR and 5x reaction buffer, 1 mM of dNTP mix, 500 nM each of the universal forward and reverse PCR primer, and Herculase II fusion DNA polymerase (Agilent Technologies, Santa Clara, CA). Cycle conditions for the first PCR were as follows: 3 min at 95°C for heat activation, 25 cycles of 30 s at 95°C, 30 s at 55°C, and 30 s at 72°C, followed by 5 min of final extension at 72°C. The PCR product was purified with AMPure beads (Agencourt Bioscience, Beverly, MA), and then 2 μL of the product was amplified using PCR for final library construction with the Nextera XT Index Kit v2 (Illumina, San Diego, USA). The cycle conditions for the second PCR were the same as those of the first PCR except that only 10 cycles were performed. The PCR product was purified with AMPure beads and the final purified product was then quantified using qPCR according to the qPCR Quantification Protocol Guide (KAPA Library Quantification kits for IlluminaSequecing platforms) and evaluated for quality using the TapeStation D1000 ScreenTape (Agilent Technologies, Waldbronn, Germany). Paired-end (2×300 bp) sequencing was performed using the MiSeq™ platform (Illumina, San Diego, USA). In the preprocessing step, the adapter trimming, read assembly and OTU clustering were performed using fastp (v.0.19.3), FLASH (v.1.2.11) and CD-HIT-OTU, respectively (Magoc and Salzberg, 2011; Li and Chang, 2017; Chen et al., 2018). The final assembly was annotated by searching against the NCBI 16S ribosomal RNA sequence (Bacteria and Archaea) database using BLASTn (v.2.4.0) (Altschul et al., 1990; Sayers et al., 2021).

### Statistical Analysis of Gut Bacterial Microbiota

Statistical analysis of the gut bacterial microbiota was conducted using MicrobiomeAnalyst (Chong et al., 2020). Low-count and low-variance filters were used under the condition of four minimum counts in samples showing the prevalence of at least 20 and 10%, respectively, based on the inter-quartile range. Data scaling was performed using total sum scaling. Relative abundance was plotted as a stacked bar plot at the phylum level. Small taxa with counts less than 1000 were merged for the relative abundance plot. Alpha diversity (species richness and evenness) was calculated using filtered data and the Shannon diversity index at the genus level was used for evenness of alpha diversity; statistical analysis of alpha diversity between Baseline and Vancomycin groups was performed using the paired t-test. Beta diversity was calculated using the distance method of the Bray-Curtis Index at the genus level, and statistical analysis was performed using PERMANOVA. Linear discriminant analysis (LDA) effect size (LEfSe) was used at the genus level with FDR-adjusted *p*-value cutoff of 0.05 and log LDA score cutoff of 2.0. Unidentified taxa were excluded from LEfSe.

### Metabolomics

Fecal, plasma, and urine untargeted metabolomics analysis was conducted using gas chromatography‐time of flight mass spectrometry (GC‐TOFMS) (LECO Corporation, St. Joseph, MI). Biospecimens were prepared using a previously published protocol with minor modifications (Fiehn, 2016). For fecal preparation, extraction solution A (3:3:2, acetonitrile: isopropanol: H_2_O) was used to extract metabolites from fecal samples using sample mass to extraction-solution volume ratio of 50 mg fecal sample/1 mL extraction solution A. For plasma and urine preparation, we used 50 μL sample/1 mL extraction solution A. For quality control (QC), extracted samples (100 µL each) were pooled to generate a QC sample, which was injected after every 10 samples. Then, each sample (400 µL) was evaporated using a nitrogen evaporator and resuspended in extraction solution B (1:1, acetonitrile: H_2_O). Next, the resuspended samples were centrifuged at 18000 x*g* for 5 min, and their supernatants were subjected to nitrogen evaporation. The dried supernatants were derivatized using 10 µL MeOX at 30°C for 90 min and 90 µL 5% FAME in *N*-methyl-*N*-(trimethylsilyl)trifluoroacetamide at 70°C for 45 min. Lastly, 1 µL of each of the prepared supernatants was injected into GC-TOFMS for untargeted metabolomics analysis.

### Statistical Analysis of Metabolome

Chromatographs were aligned using Chroma TOF 4.6 (LECO Corporation). Data processing and multivariate analysis were performed using MetaboAnalyst 4.0 after exporting the aligned chromatographs in.csv format (Chong et al., 2019). Metabolites with missing values exceeding 20% were removed, and the remaining missing values were replaced with a small value (half of the minimum positive value in the original data). Data with replaced missing values were filtered using the relative standard deviation of fecal metabolite levels greater than 30% in QC samples. Processed peak areas were normalized using sum normalization, and Pareto scaling was used for multivariate analysis. The markers were selected using a volcano plot with FDR adjusted *p*-value cutoff of less than 0.01, and fold change below or above 2.0. Statistical analysis of metabolic markers between Baseline and Vancomycin groups was performed using the Wilcoxon matched-pairs signed-rank test or paired t-test based on the Shapiro-Wilk normality test.

### Correlation Between Gut Bacterial Microbiota and Metabolome

Spearman’s rank correlation analysis was performed to explore the association between gut bacterial microbiota and metabolome using MetaboAnalyst 4.0. Sum-normalized value of the gut metabolome peak area and relative abundance of bacterial taxa at the genus level were used for the analysis. For visualization, Spearman’s rank correlation coefficients between gut bacterial microbiota and metabolome were plotted as a heatmap using Microsoft Excel (version 16.36).

### Endogenous Markers for CYP3A Activity

The concentrations of four urinary steroids (cortisol, 6β-OH-cortisol, cortisone, and 6β-OH-cortisone) and one plasma steroid (4β-OH-cholesterol) were quantified using a 7890B series gas chromatograph (Agilent Technologies, Santa Clara, CA) coupled with a 7000B series triple quadrupole mass spectrometer (Agilent Technologies, Santa Clara, CA) as described previously (Kim et al., 2018a). The ratio of 6β-OH-cortisol/cortisol and 6β-OH-cortisone/cortisone, and concentration of 4β-OH-cholesterol, were calculated and used as markers for the measurement of CYP3A activity. Statistical analysis of endogenous markers for CYP3A activity between The Baseline and Vancomycin groups was performed using the Wilcoxon matched-pairs signed-rank test or the paired t-test based on the Shapiro-Wilk normality test.

### CYP3A4 mRNA Expression

*CYP3A4* mRNA was quantified in whole blood collected from all the participants as described previously (Temesvári et al., 2012). Expression of glyceraldehyde 3-phosphate dehydrogenase (*GAPDH*) was used to normalize the expression of *CYP3A4* mRNA. Statistical analysis of *CYP3A4* mRNA expression between The Baseline and Vancomycin groups was performed using the Wilcoxon matched-pairs signed-rank test or the paired t-test based on the Shapiro-Wilk normality test.

### Prediction of Metagenomic Function: Dihydropyrimidine Dehydrogenase

Phylogenetic Investigation of Communities by Reconstruction of Unobserved States (PICRUSt2) along with the Kyoto Encyclopedia of Genes and Genomes (KEGG) database were used to predict the functional gene count of dihydropyrimidine dehydrogenase (DPD, EC 1.3.1.1) in each of the samples using the 16S rRNA gene sequences (Langille et al., 2013; Kanehisa et al., 2014; Douglas et al., 2020).

### Statistical Analysis and Plotting

Prism 9.1.0 (Graphpad, San Diego, CA) was used for statistical analysis and plotting. Statistical analysis of paired test between the Baseline and Vancomycin groups was performed using the Wilcoxon matched-pairs signed-rank test or the paired t-test based on the Shapiro-Wilk normality test.

## Results

### *16S rRNA* Gene Sequencing

The *16S rRNA* genes of 62 samples were sequenced using the MiSeq™ platform. The total number of sequence reads was 8697412. In the preprocessing step, 2 sequences with ambiguous base calls, 32956 with low-quality bases (Quality score offset 33), 385045 with chimeric reads, and 5761814 with the rest of the noise including singletons/doubletons reads were filtered. The final number of sequence reads after the preprocessing step was 2,517,595, and the min, max, median, and mean of sequence per sample were 17066, 88510, 38690, and 40606, respectively. After preprocessing, 11 subsets of samples were obtained from the 62 assessed samples to further analysis of gut bacterial microbiota.

### Gut Bacterial Microbiota

**Figure 2:**
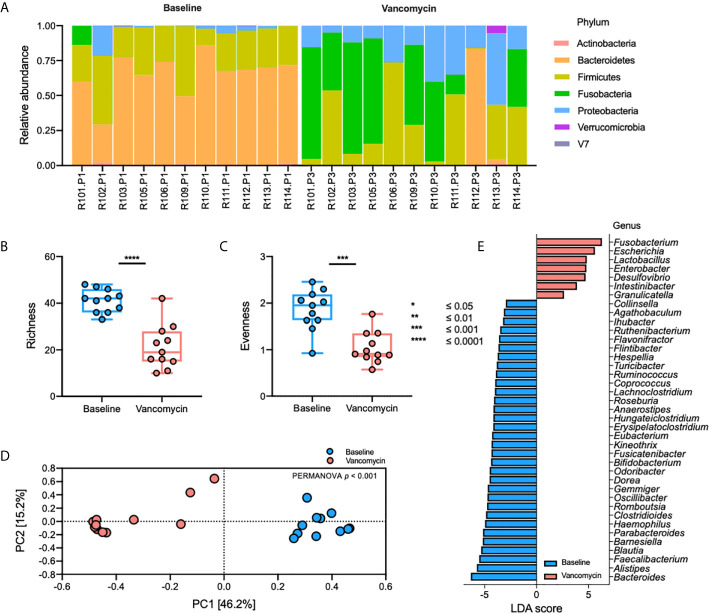
Oral vancomycin-induced alteration of gut bacterial microbiota. **(A)** Relative abundance at the phylum level, **(B)** richness and **(C)** evenness plot at the genus level, **(D)** PCoA plot derived from Bray-Curtis Index at the genus level, and **(E)** gut bacterial microbiota markers selected using LEfSe. Statistical analysis of alpha and beta diversity was performed using the paired t-test and PERMANOVA, respectively. *p ≤ 0.05; **p ≤ 0.01; ***p ≤ 0.001; ****p ≤ 0.0001.

The relative abundance plot of the gut bacterial microbiota shows a distinct composition of the gut bacterial microbiota between Baseline and Vancomycin samples (**Figure 1A**). In the Vancomycin samples, the relative abundance of nearly all genera belonging to the phyla Bacteroidetes and Firmicutes is decreased, whereas that of nearly all the genera belonging to the phyla Fusobacteria and Proteobacteria is increased (**Supplementary Table S1**). Richness (paired t-test, *p*-value < 0.0001) and evenness (paired t-test, *p*-value = 0.0005) plots show significantly higher alpha diversity in Baseline samples than in Vancomycin samples (**Figures 2B, C**). The principal coordinates analysis (PCoA) plot, generated based on the Bray-Curtis index, shows a discriminated beta diversity (PERMANOVA, *p*-value <0.001) between the Baseline and Vancomycin samples (**Figure 2D**). To discriminate the gut bacterial microbiota composition between the Baseline and Vancomycin samples, 39 genera were selected as gut bacterial microbiota markers using LEfSe (**Figure 2E**). This analysis showed that the genera *Fusobacterium*, *Escherichia*, *Lactobacillus*, *Enterobacter*, *Desulfovibrio*, *Intestinibacter*, and *Granulicatella* were increased, while the other 32 genera were decreased, after the administration of oral vancomycin (**Figure 2E**). This result demonstrates that oral vancomycin administration caused considerable dysbiosis in the human gut bacterial microbiota.

### Metabolome

**Figure 3:**
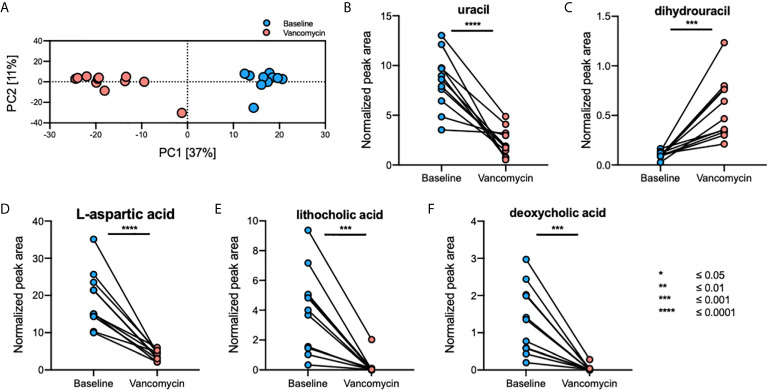
Oral vancomycin-induced alterations in the gut metabolome. **(A)** PCA plot derived from fecal metabolomics analysis and spaghetti plots derived from the normalized peak area of gut metabolic markers selected using volcano-plot analysis; **(B)** levels of uracil, **(C)** dihydrouracil, **(D)** L-aspartic acid, **(E)** lithocholic acid, and **(F)** deoxycholic acid. Statistical analysis of gut metabolic markers was performed using the Wilcoxon matched-pairs signed-rank test or the paired t-test based on the Shapiro-Wilk normality test. *p ≤ 0.05; **p ≤ 0.01; ***p ≤ 0.001; ****p ≤ 0.0001.

A total of 1645 fecal, 796 plasma, and 1255 urine metabolites were detected using GC-TOFMS. After data processing, 323 fecal, 338 plasma, and 355 urine metabolites were retained for further analysis. Principal component analysis (PCA) plots showed that the gut metabolome profile (**Figure 3A**), but not those of the plasma and urine metabolomes, differed greatly between the Baseline and Vancomycin samples (**Supplementary Figure S1**). Using a volcano-plot analysis, 42 fecal metabolites were selected as statistically significant gut metabolic markers, but no statistically significant markers were found in the plasma and urine metabolomics analysis. After filtration of noise peaks and identification using a commercially available database, five metabolites were selected and identified as gut metabolic markers. The levels of uracil (paired t-test, *p*-value < 0.0001), L-aspartic acid (paired t-test, *p*-value < 0.0001), lithocholic acid (LCA) (Wilcoxon matched-pairs signed-rank test, *p*-value = 0.001), and deoxycholic acid (DCA) (Wilcoxon matched-pairs signed-rank test, *p*-value = 0.001) were higher in Baseline samples, whereas that of dihydrouracil (DHU) (paired t-test, *p*-value = 0.0005) was higher in Vancomycin samples (**Figures 3B–F**). This result indicates that oral vancomycin exerted both direct and indirect effects on the gut metabolome by inducing dysbiosis of gut bacterial microbiota.

### Correlation Between Gut Bacterial Microbiota and Metabolome

**Figure 4:**
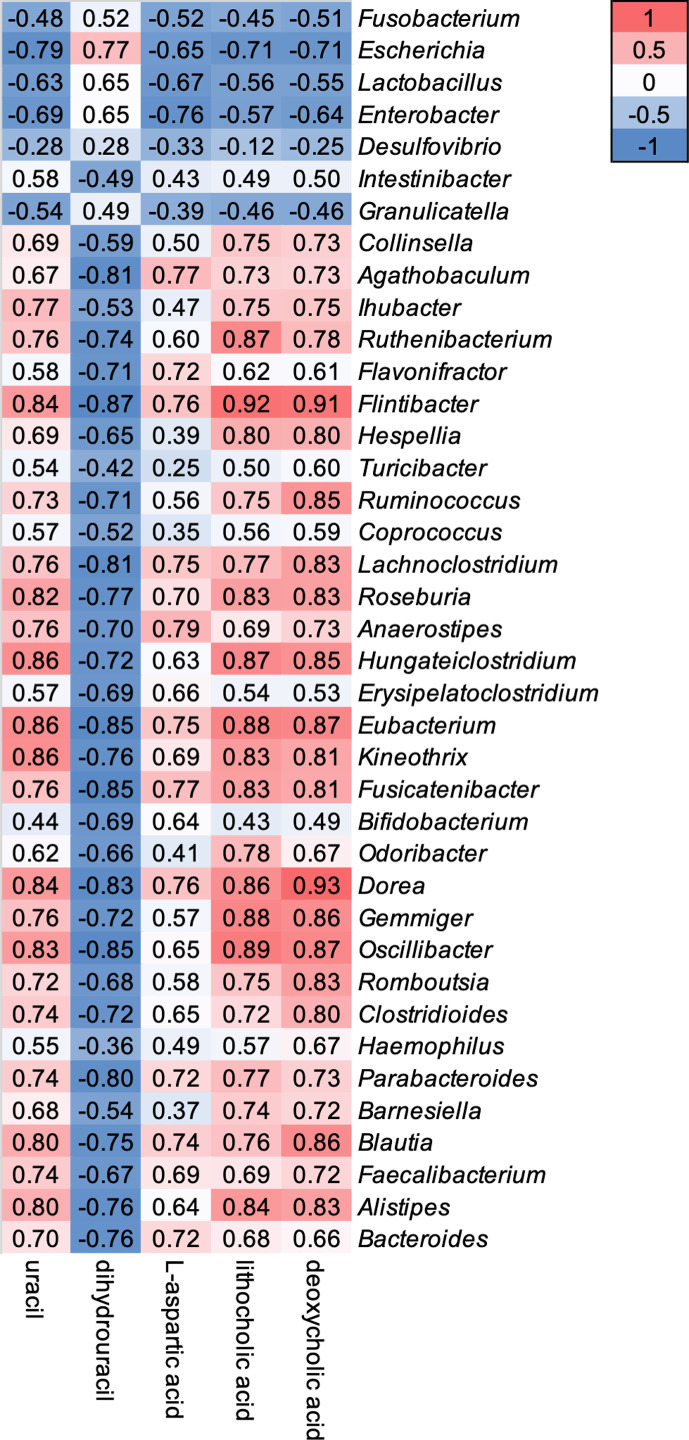
Correlations between oral vancomycin-induced alterations in the composition of the gut bacterial microbiota and gut metabolic markers. Correlation heatmap derived using Spearman’s rank correlation coefficient.

Relative abundance of 39 genera showed a weak to strong correlation with five gut metabolomic markers (**Figure 4**). Genera *Fusobacterium, Escherichia, Lactobacillus, Enterobacter, Desulfovibrio*, and *Granulicatella* showed a negative correlation with the level of uracil, L-aspartic acid, LCA, and DCA, but a positive correlation with the level of DHU (**Figure 4**). In contrast, other 33 genera showed opposite correlation with five gut metabolic markers (**Figure 4**).

### Activity of Hepatic CYP3A

DCA and LCA are endogenous ligands for the PXR, which regulates the expression of CYP3A and further enzymatic activity (Toda et al., 2009). Thus, we investigated the effect of decreased DCA and LCA levels, induced by the administration of vancomycin, on the expression of *CYP3A4* mRNA and activity of hepatic CYP3A. However, our results showed no statistical differences in the expression levels of *CYP3A4* mRNA or those of endogenous markers of CYP3A activity between the Baseline and Vancomycin samples (**Supplementary Figure S2**). This result indicates that a significant decrease in the levels of intestinal secondary bile acids, DCA and LCA, did not significantly affect the activity of hepatic CYP3A.

### Predicted DPD Functional Gene Count

Predicted functional gene count of DPD was significantly higher (Wilcoxon matched-pairs signed-rank test, *p*-value = 0.0137) in Vancomycin samples compared to that of Baseline samples (**Supplementary Figure S3**).

## Discussion

Antibiotics discovered during the antibiotics era have greatly increased human life expectancy; however, these compounds are also associated with diseases (Adedeji, 2016), and among the incidental consequences of antibiotics usage, we found the dysbiosis of the human gut microbiota (Vrieze et al., 2014; Isaac et al., 2017). Therefore, in this study, we investigated the effect of oral vancomycin, a commonly used antibiotic, on gut bacterial microbiota and the metabolome in biospecimens collected from healthy men. Our study was conducted to gain a broad overview of how antibiotic-induced dysbiosis of human-gut bacterial microbiota may impact drug metabolism and further human health. We performed 16S rRNA gene sequencing and metabolomics analyses using biospecimens collected before and after the administration of oral vancomycin. Our results show significantly decreased relative abundance of the genera belonging to the phyla Bacteroidetes and Firmicutes, and increased relative abundance of the genera from the Proteobacteria and Fusobacteria phyla, following the administration of oral vancomycin (**Figure 2** and **Supplementary Table S1**). Additionally, the levels of uracil, L-aspartic acid, LCA, and DCA were higher in Baseline samples, whereas that of DHU was higher in Vancomycin samples (**Figure 3**). These results show that the composition of both gut bacterial microbiota and metabolome underwent significant changes after oral administration of vancomycin. This result was consistent with those reported in previous studies (Vrieze et al., 2014; Sunwoo et al., 2020). In addition, Vrieze et al. have shown that oral vancomycin administration induced alteration of the gut microbiota in obese participants (Vrieze et al., 2014), and they also observed significantly decreased levels of fecal secondary bile acids such as LCA and DCA after the administration of oral vancomycin. In agreement with Vrieze et al., in a previous study, we have reported a decreased relative abundance of Bacteroidetes and increased relative abundance of Proteobacteria in healthy participants after the administration of oral vancomycin (Sunwoo et al., 2020). The results of these previous studies using different cohorts, and those of our present study, show that regardless of the health status of the participants, administration of oral vancomycin exerted similar effects on the gut microbiota composition. These findings indicate that oral vancomycin-induced alterations of gut bacterial microbiota may exert disease-specific effects on different cohorts by significantly decreasing the dominant phyla Bacteroidetes and Firmicutes. To prevent the negative effects of orally administered vancomycin, this effect should be investigated in cohorts with diseases reportedly associated with the phyla, Bacteroidetes and Firmicutes.

DPD is an essential enzyme responsible for the degradation of pyrimidine and 5-fluorouracil (5-FU), which is a pyrimidine analog used as a drug against cancer and extensively utilized to manage malignant tumors such as those of the colon, breast, and skin (Diasio and Harris, 1989; Kubota, 2003). When administered, more than 80% of 5-FU undergoes catabolism by DPD and is degraded; thus, the activity of DPD is essential for regulating the PK of 5-FU (Heggie et al., 1987). Here, we observed decreased levels of fecal uracil and increased levels of fecal dihydrouracil after the administration of oral vancomycin (**Figure 3**), which were strongly correlated with increased and decreased relative abundance of genera *Escherichia, Lactobacillus* and *Enterobacter*, respectively (**Figure 4**). In addition, we observed significantly higher levels of predicted functional gene counts of DPD in Vancomycin samples compared to that of Baseline samples when the functional gene count was predicted using PICRUSt2 with KEGG pathway (**Supplementary Figure S3**). Therefore, we speculated that the PK of oral 5-FU may be significantly affected by oral vancomycin-induced increase in the relative abundance of genera *Escherichia, Lactobacillus* and *Enterobacte*, which may further enhance the enzymatic activity of 5-FU dehydrogenation in the gastrointestinal tract. Additionally, consistent with our results, Yuan et al. have shown an increased gut bacterial abundance of the genera *Escherichia* and *Enterobacter* in a colorectal cancer mouse model after treatment with 5-FU and a cocktail of antibiotics including vancomycin (Yuan et al., 2018). Accordingly, the increased abundance of the two genera was associated with diminished antitumor efficacy of 5-FU in the mice model. Thus, the interaction of 5-FU with *Escherichia* and *Enterobacter* microorganisms should be further confirmed *in vitro* to be clinically applicable. For example, the potential interactions of altered *Escherichia* and *Enterobacter* genera levels induced by oral vancomycin should be considered when prescribing pyrimidine analogs (Takimoto et al., 1996).

Previous studies have demonstrated that gut microbiota regulate the levels of gut bile acids *via* primary bile acid metabolism or by the mediation of bile acid synthesis (Ridlon et al., 2006; Kakiyama et al., 2013; Sayin et al., 2013). Among the various classes of secondary bile acids, lithocholic acid (LCA) and deoxycholic acid (DCA) are metabolized from chenodeoxycholic acid (CDCA) and cholic acid (CA), respectively, by the genus *Clostridioides* (Ridlon et al., 2006). Additionally, LCA enhances the activity of hepatic Cyp3a11, a murine homolog to human CYP3A4, by activating PXR in mice (Ridlon et al., 2006). In agreement with these previous studies, our results indicate that oral vancomycin induced decreased levels of LCA and DCA, and relative abundance of the genus *Clostridioides* (**Figures 2E** and **3E, F**). Moreover, we observed a strong positive correlation between the altered levels of LCA and DCA, and changes in the relative abundance of *Clostridioides*, after the administration of oral vancomycin (**Figure 4**). Thus, we investigated whether decreased levels of LCA and altered relative abundance of *Clostridioides*, induced by oral vancomycin, affected the expression of CYP3A4 and activity of hepatic CYP3A. However, we did not observe any significant changes in the expression of CYP3A4 or in the activity of hepatic CYP3A after the administration of oral vancomycin (**Supplementary Figure S2**). This negative result may have resulted from the relatively short duration of treatment with oral vancomycin. Additionally, because LCA enhances CYP3A activity, the decreased level of LCA induced by oral vancomycin may have a negligible effect on the activity of hepatic CYP3A. Nevertheless, the long-term effects of oral vancomycin on CYP3A activity, understood as the time span necessary for CYP3A hepatic activity to be affected by endogenous secondary bile acids through PXR activation, should be further investigated *in vivo* and *in vitro* to prevent the drug-drug interactions with compounds that are mainly metabolized by CYP3A.

A limitation of this study is that no samples were collected after the washout period of oral metformin but before administration of oral vancomycin, and we therefore cannot evaluate the potential effect of oral metformin on the gut bacterial microbiota. Four months of oral metformin treatment increased the genus *Escherichia* and decreased the genus *Intestinibacter* relative abundances in 22 individuals with treatment-naïve type 2 diabetes (Wu et al., 2017). Similarly, the severity of gastrointestinal side effects are associated with increased relative abundance of *Escherichia-Shigella* spp. after oral metformin treatment for 7 days in 18 healthy individuals (Elbere et al., 2018). These reports suggest that oral metformin on alters the gut bacterial microbiota both in long-term treated type 2 diabetes patients and relatively short-term treated healthy subjects. In our single-arm study, we included 5 days of washout period after administration of oral metformin to eliminate the potential pharmacological effects of the drug on the subjects before starting the second phase of the clinical study, but the effect of the washout period on the gut bacterial microbiota was not evaluated. However, we speculate that the effect of 4 days of oral metformin treatment on the results of this study would not be significant because we observed a consistent effect of oral vancomycin on decreasing and increasing the relative abundance of Bacteroidetes and Proteobacteria, respectively, without the involvement of oral metformin in a previous study (Sunwoo et al., 2020).

In conclusion, our present study demonstrated that oral vancomycin induced alterations in the composition of gut bacterial microbiota and metabolome. Correlation analysis of our two datasets showed that alterations in the composition of gut microbiota, induced by oral vancomycin, potentially affected the systemic activity of dihydropyrimidine dehydrogenase. This correlation should be further investigated to define the effects of gut microbiota on drug-metabolizing enzymes. These future studies could take us one step closer to achieving personalized therapy.

## Data Availability Statement

The original contributions presented in the study are publicly available. This data can be found here: NCBI repository, accession numbers: MW768153-MW768700.

## Ethics Statement

The studies involving human participants were reviewed and approved by Seoul National University Bundang Hospital Institutional Review Board. The patients/participants provided their written informed consent to participate in this study.

## Author Contributions

AK, EK, Ja-YC, and Jo-YC designed the project. AK carried out the experiment with support from YL and SJ. AK wrote the manuscript with support from EK, SJ, Ja-YC and Jo-YC. Jo-YC supervised the project. All authors contributed to the article and approved the submitted version.

## Funding

This work was supported by grants (No. NRF-2016M3A9B6902851 and No. NRF-2018R1D1A1B07044406) from the National Research Foundation of Korea (NRF) funded by the Korean government (MSIT).

## Conflict of Interest

The authors declare that the research was conducted in the absence of any commercial or financial relationships that could be construed as a potential conflict of interest.
