# Supplementary material for: Assessment of Oral Vancomycin-Induced Alterations in Gut Bacterial Microbiota and Metabolome of Healthy Men
Source: Front Cell Infect Microbiol. 2021 May 27;11:629438. doi: 10.3389/fcimb.2021.629438 (PMC8190408; doi:10.3389/fcimb.2021.629438)
Supplement: Supplementary file 1 [file DataSheet_1.pdf]

Supplementary Material

1 Supplementary Figures and Tables

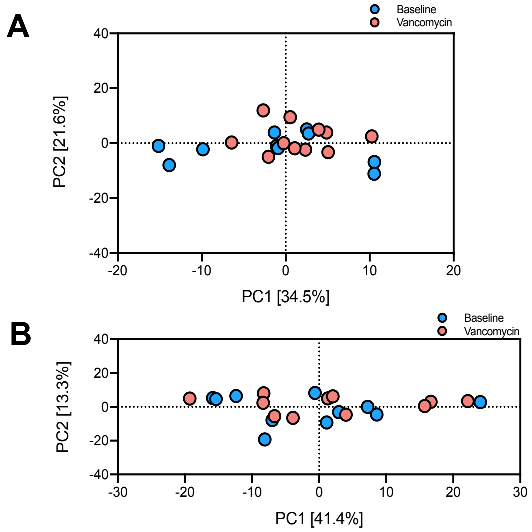

Supplementary Figure 1. Oral vancomycin induced alteration of plasma and urine metabolome. PCA plot derived from **(A)** plasma and **(B)** urine metabolomics analysis.

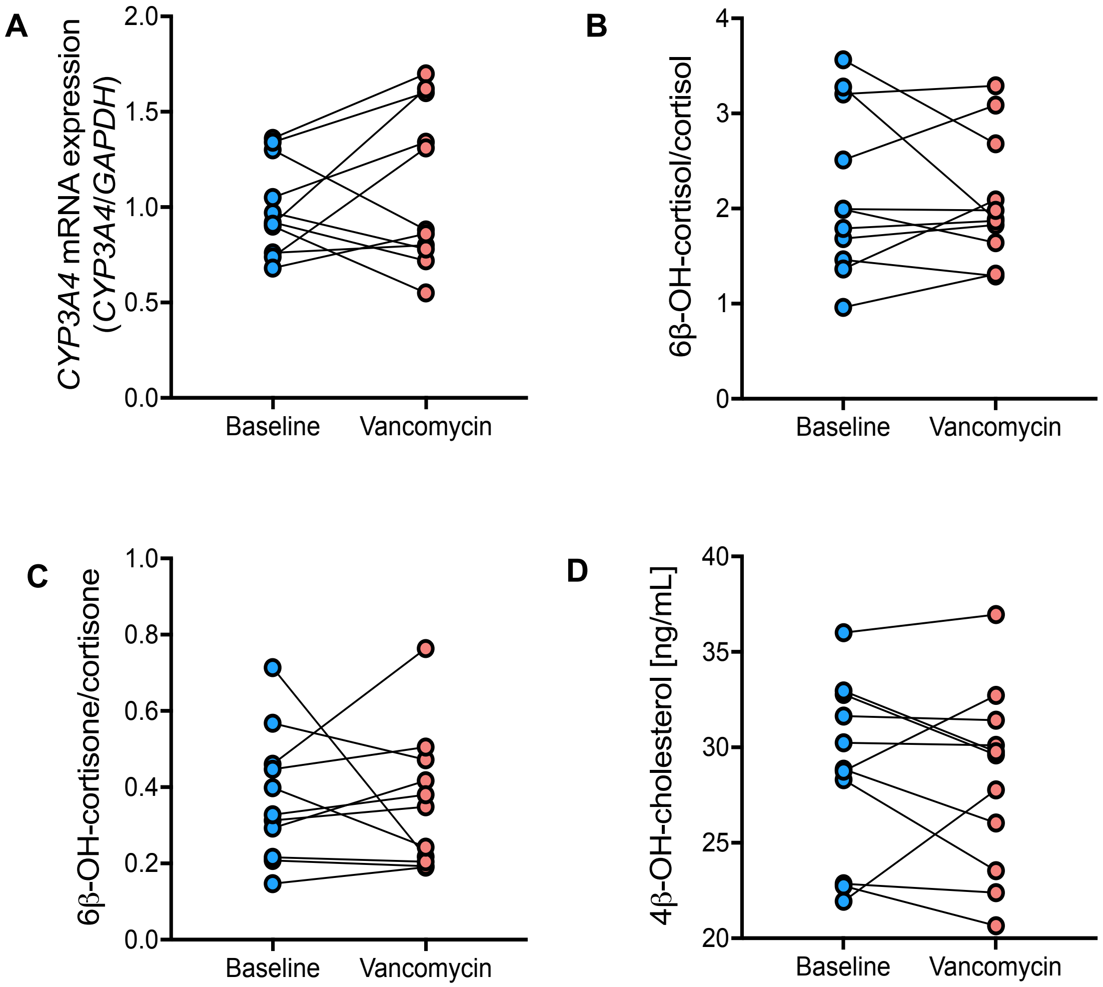

Supplementary Figure 2. Effect of oral vancomycin on the activity of CYP3A. Spaghetti plot derived from **(A)** *CYP3A4* mRNA expression, **(B)** 6β-OH-cortisol/cortisol, **(C)** 6β-OH-cortisone/cortisone, and **(D)** 4β-OH-cholesterol of Baseline and Vancomycin. Statistical analysis was performed using the Wilcoxon matched-pairs signed-rank test or paired t-test based on the Shapiro-Wilk normality test.

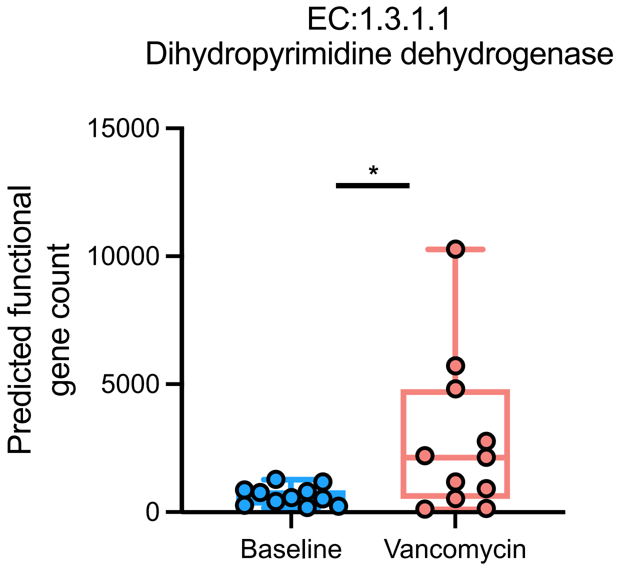

Supplementary Figure 3. Predicted functional gene count of DPD in Baseline and Vancomycin. Significantly higher (Wilcoxon matched-pairs signed-rank test, *p*-value = 0.0137) predicted functional gene count of DPD was observed in Vancomycin compared to that of Baseline. Statistical analysis was performed using the Wilcoxon matched-pairs signed-rank test. $*p \leq 0.05$; $**p \leq 0.01$; $***p \leq 0.001$; $****p \leq 0.0001$.

Supplementary Table 1. Gut bacterial microbiota markers selected from LEfSe analysis.

**Phylum**	**Genus**	**Mean abundance in Baseline**	**Mean abundance in Vancomycin**	**Fold change of Baseline/ Vancomycin**	**FDR adjusted *p*-value**	**LDA score**
Fusobacteria	*Fusobacterium*	125390	4059800	0.0309	0.0119	6.29
Proteobacteria	*Escherichia*	50858	876650	0.0580	0.0016	5.62
Firmicutes	*Lactobacillus*	302.89	136550	0.0022	0.0011	4.83
Proteobacteria	*Enterobacter*	994.57	126860	0.0078	0.0004	4.8
Proteobacteria	*Desulfovibrio*	5197.4	110220	0.0472	0.0351	4.72
Firmicutes	*Intestinibacter*	5413.4	20881	0.2593	0.0481	3.89
Firmicutes	*Granulicatella*	168.24	1035.9	0.1624	0.0189	2.64
Actinobacteria	*Collinsella*	13985	12149	1.1511	0.0042	-2.96
Firmicutes	*Agathobaculum*	2832	13.054	216.9450	0.0003	-3.15
Firmicutes	*Ihubacter*	3753.8	391.36	9.5917	0.0021	-3.23
Firmicutes	*Ruthenibacterium*	6700.8	187.34	35.7681	0.0013	-3.51
Firmicutes	*Flavonifractor*	8385.2	13.054	642.3472	0.0004	-3.62
Firmicutes	*Flintibacter*	9078.8	43.485	208.7800	0.0003	-3.66
Firmicutes	*Hespellia*	11350	347.88	32.6262	0.0047	-3.74
Firmicutes	*Turicibacter*	14583	1454.5	10.0261	0.0149	-3.82
Firmicutes	*Ruminococcus*	16334	230.83	70.7620	0.0026	-3.91
Firmicutes	*Coprococcus*	19570	443.93	44.0835	0.0038	-3.98
Firmicutes	*Lachnoclostridium*	20269	56.89	356.2841	0.0003	-4

Firmicutes	*Roseburia*	24743	0		0.0007	-4.09
Firmicutes	*Anaerostipes*	27419	156.56	175.1341	0.0003	-4.13
Firmicutes	*Hungateiclostridium*	28449	43.485	654.2256	0.0012	-4.15
Firmicutes	*Erysipelatoclostridium*	28760	21.133	1360.9047	0.0088	-4.16
Firmicutes	*Eubacterium*	38180	1187.5	32.1516	0.0003	-4.27
Firmicutes	*Kineothrix*	41672	331.92	125.5483	0.0003	-4.32
Firmicutes	*Fusicatenibacter*	44120	0		0.0003	-4.34
Actinobacteria	*Bifidobacterium*	47447	281.78	168.3831	0.0016	-4.37
Bacteroidetes	*Odoribacter*	64560	1703.5	37.8984	0.0125	-4.5
Firmicutes	*Dorea*	66101	217.42	304.0245	0.0003	-4.52
Firmicutes	*Gemmiger*	100020	408.09	245.0930	0.0007	-4.7
Firmicutes	*Oscillibacter*	106690	1004.6	106.2015	0.0003	-4.72
Firmicutes	*Romboutsia*	121190	9121.8	13.2858	0.0017	-4.75
Firmicutes	*Clostridioides*	141310	301.89	468.0844	0.0003	-4.85
Proteobacteria	*Haemophilus*	182560	2594.9	70.3534	0.0158	-4.95
Bacteroidetes	*Parabacteroides*	277980	444.63	625.1940	0.0003	-5.14
Bacteroidetes	*Barnesiella*	299090	3913.6	76.4232	0.0107	-5.17
Firmicutes	*Blautia*	439370	17616	24.9415	0.0003	-5.32
Firmicutes	*Faecalibacterium*	661050	15290	43.2341	0.0003	-5.51
Bacteroidetes	*Alistipes*	1115500	12013	92.8577	0.0005	-5.74
Bacteroidetes	*Bacteroides*	4003900	2693.3	1486.6149	0.0003	-6.3
